# Prolonged Surveillance in Inverted Papilloma Reveals Delayed Recurrence and Lack of Benefit to Frozen Section

**DOI:** 10.1177/19458924241305658

**Published:** 2024-12-20

**Authors:** Kush Panara, Alan D. Workman, David K. Lerner, Charles C. L. Tong, Jadyn Wilensky, Jennifer E. Douglas, Nithin D. Adappa, James N. Palmer, Michael A. Kohanski

**Affiliations:** 1 Department of Otorhinolaryngology, 6572 University of Pennsylvania, Philadelphia, Pennsylvania; 2 Department of Otolaryngology, University of Miami, Miami, Florida; 3 Department of Otolaryngology, Lenox Hill Hospital, New York, New York; *Current affiliation: Department of Otolaryngology, Harvard Medical School, 25 Shattuck St, Boston, MA, 02115, USA.

**Keywords:** inverted papilloma, sinonasal malignancy, frozen section, sinonasal surgery, recurrence

## Abstract

**Background:**

To reduce recurrence rates of inverted papilloma (IP), some have argued for the use of intraoperative frozen margins; results remain mixed and studies critically lack lengthy surveillance periods.

**Objective:**

We aim to elucidate the impact of prolonged surveillance and intraoperative frozen margins on IP recurrence.

**Methods:**

This is a retrospective analysis of patients who underwent resection of IP at a tertiary care center over a 10-year period from 2008 to 2018 followed by subsequent surveillance. Patient demographics, tumor and operative characteristics, and recurrences were analyzed.

**Results:**

Our analysis includes 199 patients, with 37 recurrences and an average recurrence time of 44.4 months; 57% of patients received intraoperative frozen sections and recurrence rates were similar between those who received frozen sections and those who did not (20.1% vs 15.5%, *P* = .36). Patients with recurrences within 5 years of surgery were more likely to have received frozen sections than those with recurrences beyond 5 years (*P* < .01). There was no difference in surgical approach or extent of disease in those who received frozen margins. Patients that received frozen sections were more likely to have multiple sites of attachment (56.5% vs 38.1%, *P* = .01) and persistent disease following a previous resection at an outside institution (67.0% vs 44.0%, *P* = .001).

**Conclusion:**

Our average time to recurrence was 44.4 months, significantly longer than surveillance times reported in the literature, indicating that longer periods of surveillance are necessary to capture late recurrences. Our analysis is the first and largest American cohort to look at IP resection in a standardized fashion and find that recurrence rates are similar between patients receiving frozen sections or not.

## Introduction

Inverted papilloma (IP) is the most common benign sinonasal tumor, characterized by its ability for inward growth into the underlying stroma and bone. It has a tendency for recurrence and potential for malignant transformation. Current primary treatment is attachment-oriented gross tumor resection and long-term surveillance with serial clinical evaluations to assess for recurrence.

Despite advancements in endoscopic and surgical techniques, the recurrence rates of IP remain high, up to 21%.^1,2^ In an effort to reduce recurrence rates, some have advocated for the use of intraoperative frozen sections to obtain clear margins and to guide extent of resection.^3^ However, results remain mixed, and current studies suffer from small or heterogenous samples and critically lack lengthy surveillance periods. We aim to elucidate the impact of intraoperative frozen margins on IP recurrence in a single institution with standardized operative technique.

## Methods

This study was approved by the University of Pennsylvania Institutional Review Board. A retrospective analysis was performed of patients who underwent resection of IP by 2 senior authors (NA and JP) at a tertiary care center over a 10-year period from 2008 to 2018 followed by subsequent surveillance. All cases underwent standardized operative management, which entailed complete surgical removal of IP along with drilling of bony attachment site when identified. Patients with concurrent tumors of other pathologies (exophytic or oncocytic types) were excluded. Patient demographics, tumor and operative characteristics, and use of intraoperative frozen section were analyzed. Recurrence was defined as diagnosis of IP at the same site as the prior resection following complete surgical excision at our institution. Patients who underwent primary surgery at an outside institution were not considered to have true recurrences in our analysis. Time to recurrence was measured from time of initial surgery to pathologic diagnosis of the recurrence.

Statistics were performed using Stata (version 13, StataCorp). Univariate analysis was performed using χ^2^ or *t* tests to determine a difference in categorical risk factors between patients who received frozen sections and those who did not. A *P* value of <.05 was considered statistically significant and all tests were 2-tailed.

## Results

Our analysis includes 199 patients with confirmed diagnosis of IP who underwent surgical resection at our institution from 2008 to 2018. Mean follow-up was 53.9 months (range 3-180 months). Demographics including age, sex, and race are included in Table 1. There were 37 recurrences (17.1%) with an average recurrence time of 44.4 months. One hundred and fifteen patients (57.8%) had intraoperative frozen sections and recurrence rates were similar between those who received frozen sections and those who did not (20.1% vs 15.5%, *P* = .36, Table 2). Patients with recurrences within 5 years of surgery were more likely to have received frozen sections than those with recurrences beyond 5 years (*P* < .01).

**Table 1.** Patient Demographics.

Demographics	No frozen margins	Frozen margins
Number of patients	84	115
Age (years)	60.0 ± 12.3	55.3 ± 13.6
Male sex	59 (70.2%)	73 (63.5%)
Caucasian race	61 (72.6%)	87 (75.7%)

**Table 2.** Surgical Characteristics and Outcomes Between Patients Receiving Frozen Sections and Those Who Did Not.

Surgical characteristics	No frozen margins	Frozen margins	*P* value
History of prior surgery for IP	65 (77.4%)	73 (63.5%)	.**04**
Endoscopic only approach	64 (76.2%)	85 (73.9%)	.35
Extrasinus extension	16 (19.0%)	14 (12.2%)	.23
Multiple sinuses involved	15 (17.9%)	30 (26.1%)	.23
Multiple sites of attachment	32 (38.1%)	65 (56.5%)	.**01**
Disease type			.**001**
Primary disease	47 (56.0%)	38 (33.0%)	
Persistent/recurrent disease	37 (44.0%)	77 (67.0%)	
Pathology			.42
*No dysplasia*	56 (66.7%)	77 (67.0%)	
*Dysplasia/SCCa*	28 (33.3%)	38 (33.0%)	
Number of surgeries			.24
*1*	64 (76.2%)	84 (73.0%)	
*2*	16 (19.0%)	18 (15.7%)	
*3+*	4 (4.8%)	13 (11.3%)	
CSF leak during surgery	8 (9.5%)	8 (7.0%)	.6
Outcomes			
Recurrence	13 (15.5%)	24 (20.1%)	.36
Time to recurrence (months)	50.8 ± 31.3	34.1 ± 25.3	.11
Chemotherapy/adjuvant radiation	4 (4.8%)	13 (11.3%)	.13

Abbreviations: IP, inverted papilloma; SCCa, squamous cell carcinoma; CSF, cerebrospinal fluid. Bold represents significant values *p* < 0.05.

There was no difference in surgical approach, extent of disease, number of sinuses involved, or severity of pathology in those who received frozen margins compared to those who did not. Patients that received frozen sections were more likely to have multiple sites of attachment (56.5% vs 38.1%, *P* = .01) and persistent disease following a previous resection at an outside institution (67.0% vs 44.0%, *P* = .001, Table 2).

## Discussion

Minimizing recurrence rate remains a challenge in the treatment of sinonasal IP. Complete resection of the tumor along with drilling the underlying bony attachment has been associated with reduced recurrence rates^4^ and is the technique employed at our institution. The recurrence rate seen here of 17.1% is consistent with previous reports in the literature, particularly for tertiary treatment centers with bias toward high-risk, multirecurrent, or revision cases.^5–7^

Given the propensity for IP to grow submucosally and into the underlying bone, utilizing intraoperative frozen sections was described by Thaler et al as early as 1999 for suspect cases.^8^ More recently, Healy et al were the first to evaluate the impact of frozen sections on recurrence rates, finding no benefit of negative margins on frozen section on recurrence rates.^4^ In contrast, Miglani et al demonstrated 100% negative predictive value and 100% positive predictive value of frozen section concordance with final pathology. However, their cohort of 22 patients had zero recurrences and no control group, limiting evaluations on the impact on recurrence rates. A recent systematic review by Trent et al^9^ included 258 patients, the largest to date, and found obtaining negative margins on frozen section significantly reduces recurrence rates compared to not using frozen sections. However, the review includes patients from 7 different institutions without stratifying by the different operative techniques utilized with frozen section recurrence, potentially confounding results. Most recently, Glikson et al^10^ evaluated 220 patients at 4 European institutions who underwent attachment-oriented surgery for IP. Utilization of frozen sections had no impact on recurrence rates, with average time to recurrence being approximately 18 months.

Our analysis is the first and largest cohort in the United States to look at IPs resected in a standardized fashion and find that recurrence rates are similar between patients receiving frozen sections and those who did not undergo frozen section. Interestingly, although patients with multiple attachment sites had similar recurrence rates as patients with unilateral attachment, those with multifocal attachment had a higher incidence of frozen sections sent for margins. Prior work has shown that patients with multifocal attachment are 3.5 times more likely to have recurrence.^5^ Given that multifocal attachment may indicate more aggressive disease, frozen sections may aid surgeons in identifying all attachment sites and achieving complete resection. Based on our data, our institution does not routinely employ frozen section for IP with unifocal attachment, but its utility in higher risk disease has yet to be determined.

Importantly, the mean follow-up length of 53.9 months in our cohort revealed an average time to tumor recurrence of 44.4 months, significantly longer than times reported in the literature, indicating that longer periods of surveillance are necessary. Although the prior analysis by Glikson et al^10^ had a similarly long follow-up period of 49 months, they reported a mean time to recurrence of 18 months. The striking contrast in recurrence rates between our studies raises the question of a difference in underlying pathology, potentially related to patient demographics or geographic predisposition between the European and American patients.

Most of the work associating risk factors with recurrence rate has looked at the short-term recurrence within 2 years.^11^ The exact etiology of late recurrence is unclear, and we do not know if this is the natural progression of the disease that may be related to the ability for IP to involve surrounding tissue and bone or if recurrence may be triggered by an unknown inciting incident such as from an inflammatory response. Our institution's current surveillance protocol for IP includes post-treatment endoscopy every 3 months for the first year and then every 6 months for years 2 to 5. This is followed by yearly endoscopy thereafter with more frequent follow-up and biopsies for any suspicious lesions or concerning symptoms.

A major limitation of this study, given its retrospective nature, was the inability to derive what led to frozen sections being obtained and if they changed intraoperative management. While we do show that the overall extent of tumor was similar between cohorts, it is unclear if positive frozen sections led to further resection and if that had an impact on recurrence rate. A prospective analysis is needed to further evaluate the role of frozen sections.

## Conclusion

Frozen section is employed by some in an attempt to reduce the recurrence rate of IP. In our study, we find that frozen section had no impact on recurrence rates and may not be necessary for lower risk patients. Furthermore**,** in our cohort with extended surveillance periods, we find a propensity for delayed recurrence with an average time to recurrence of 44 months. To capture late recurrence, we recommend extended surveillance for patients with semiannual surveillance through the first 5 followed by at least yearly endoscopy thereafter.
